# Renu Khanna: giving women a voice in health and health care

**DOI:** 10.2471/BLT.21.030721

**Published:** 2021-07-01

## Abstract

Renu Khanna talks to Andréia Azevedo Soares about implementing feminist-inspired health interventions and encouraging women to make themselves heard on matters of health policy.

*Q: How did your engagement with women’s health and rights begin?*

A: I grew up in a very liberal household and was encouraged by my father to be independent and make my own decisions. So, I was not very aware of the challenges faced by other women growing up. I pursued studies in economics and eventually did an MBA (master’s in business administration) but even before I finished my degree, I knew that I was going to be a misfit in the corporate world. So I found a job with the Voluntary Health Association of India where I worked with people who were committed to addressing the health issues faced by the rural poor and to alleviating poverty.

The real “light bulb” moment came in 1984 when I was invited by a women’s cooperative in Jabalpur in central India. The women, who were very poor and, for the most part, illiterate, produced and sold spices. I spent the first day working with them on their business and in the evening visited the home of their leader. I will never forget it. It was this dilapidated hut in the middle of a slum. The woman’s husband had tuberculosis and was lying on a charpoy (a kind of bed) outside the hut, and the woman, who was the breadwinner for the family, was going about her business, in the most matter of fact way – with the utmost dignity, and without a shred of self-pity. I was so moved by her courage and resilience. My commitment to women’s rights was born from that experience. Around the same time, we co-founded SAHAJ and our very first project was with women waste pickers in the city.

*Q: What is SAHAJ?*

A: SAHAJ-Society for Health Alternatives, to give the organization its full name, is a non-profit committed to supporting and promoting the health and education of children, adolescents and women. We work with stakeholders to ensure access to quality health care and other public services. We also concentrate on policy advocacy, maintaining an intersectoral perspective to reflect the way in which women’s health interconnects with other aspects of their lives, including their work.

*Q: Can you say more about how those interconnections are manifested?*

A: In the mid-1980s, we were invited by an organization working in a tribal area in western India. The focus was on watershed management and improved agricultural techniques. So, on the face of it nothing to do with women or women’s health. But we got into conversations with women who were collecting wood and other biomass for their cooking needs. We introduced them to a new kind of smokeless cooking stove and in training them how to use them we learned about the other challenges they were facing. The area was going through a very bad drought and the women were becoming anaemic and further malnourished. The more we talked, the more it became apparent that they were living with all kinds of health issues, ranging from backache and depression, to menstrual problems, urinary and genital infections, and pregnancy-related issues. Inevitably, the subject of health services, or the lack of health services came up and we started thinking about what we could do to help. A young feminist doctor from the UK (United Kingdom of Great Britain and Northern Ireland), who was volunteering in India at that time, helped us design a feminist-inspired maternal health programme that could be implemented in a setting essentially without services. That was when we started training local women to deliver maternal health care.

“We seek to strengthen women’s voices, training them to generate data, produce reports and apply evidence.”

*Q: How did you go about that task?*

A: We started with a group of traditional midwives and local leaders and ran training sessions using the self-examination methodology set out in *Our Bodies, Ourselves*, the Boston women's collective book that was published in the early 1970s. The participants were encouraged to examine themselves, including their genitalia, using a speculum, a mirror and a flashlight. Every month we would meet with the women for two days so that we could share challenges encountered and solutions found. The women we trained in that way became agents of change, spreading the word to other women. We called them the "barefoot gynaecologists".

*Q: What was the reaction from the local health authorities?*

A: One day the district health officer came and said that what these women were doing was irresponsible. We said if government doctors and nurses were present, the women would not have to do what they were doing. We invited him to talk with the health workers we had trained and see if what they were doing was safe and within the limits and boundaries of what they could do. He grilled them with all kinds of technical questions, and they were able to answer him with utmost confidence and accuracy. He went away extremely impressed, but also with a better idea of what the community’s health needs were.

*Q: When did you first become aware of Our Bodies, Ourselves?*

A: In the late 70s. I remember one of my colleagues at the Voluntary Health Association of India raving about a Rural Women's Group in South India that was translating and adapting the book. I went straight to the library and found a copy. I was absolutely fascinated, not just by the idea of women coming together to share their thoughts and feelings about their lives and their bodies, but also being able to use that process to produce this medically sound book. One of the first publishing projects that a group of us undertook in the 1980s was to translate a book produced by rural women called *Sharir Ki Jankari* (knowledge about our bodies) into Gujarati. And later, in the early 90s, our nationwide feminist health research collective, Shodhini, produced a book based on the barefoot gynaecologists’ initiative, called *Touch me, touch-me-not: women, plants and healing.*

*Q: To what extent was it necessary to adapt material deriving from a western feminist perspective to the Indian context?*

A: (laughing) Well, we had to tone some of it down a little! I remember the *Sharir Ki Jankari* book had little flaps over some of the drawing to avoid giving offence. People could lift them if they were curious about the male and female genitalia. But I think it is important to emphasize that the material was about connecting women with themselves as much as with their bodies; it was about supporting them in establishing their identities and encouraging them to find their own voice and place in society. I remember the first time I organized a training programme, I asked the participants some questions about themselves: what they liked to do, what their preferences were. The response was an awkward silence. Because nobody had ever asked them such a thing! And, indeed, the prevailing cultural norms demanded that they *not* express themselves. This was such a profound learning experience for us. We asked ourselves, “If women cannot talk about themselves, barely recognize themselves as individuals, how can they embrace the notion of individual rights, including the right to health?” So, a big focus of our work at SAHAJ is making sure women do express themselves and that their voices are heard.

“Policy-makers need to open their doors and ears to community stakeholders.”

*Q: Which strategies do you use to achieve those aims?*

A: We start by listening with respect and creating spaces in which women feel that they can talk and be heard. This activity is often neglected in the dominant paradigms of health research, which tend to put the stress on biomedical and epidemiological data and quantitative research, discounting qualitative health research based on women’s testimonies and anecdotes. Women’s voices are also absent from policy discussions. Who gets access to the government of India or the state health departments? International NGOs and global universities’ researchers. Not rural or tribal women’s collectives and community-based organizations. So, we make sure these groups are heard, then amplify ground-level voices and ensure that they reach policy-making arenas by leveraging state, national and global coalitions. We also document our work, sharing lessons learnt and insights, often using social media. Finally, we seek to strengthen women’s voices, training them to generate data, and produce reports and to use that evidence in their conversations with health system stakeholders.

*Q: What impact has this had?*

A: The impact ranges from the individual to the collective. For example, we work with adolescent girls on gender and sexual reproductive health and rights and we see tremendous improvements in girls’ ability to express themselves regarding their desires for higher education and employment, as well as in their ability to negotiate within their families, and with their husbands when they get married. At the collective level, women’s organizations supported by us have been able to successfully negotiate for increased staff at the primary health centres and for enhanced skills training for frontline health workers. They have also been able to better make their case in village council meetings, for example demanding a roster of blood donors or emergency vehicles in case of maternal health emergencies. Of course, there is a limit to what community participation can achieve, and resources must come from the State, especially in the ongoing pandemic which is having a devastating impact on women’s lives and access to health care.

*Q: What has been the impact of the pandemic so far?*

A: In the lockdown all routine health services, including maternal health, came to an almost complete stop because resources were diverted to screening, testing and treatment for COVID-19. But women's reproductive cycles don't stop just because there's a pandemic. So reproductive health services need to be maintained and should be prioritized when it comes to building back better. Attention should also be given to reducing violence against women and ensuring provision of mental health services. Of course, short term, massively increased government support is needed to ensure access to COVID-related vaccines, therapeutics, ventilators, and oxygen. Civil society is very strong in India, but the situation is really overwhelming now, and government needs to come forward. Longer term, policy-makers need to open their doors and ears to community stakeholders and give plenty of scope for local autonomous action and decentralized planning to address health inequities, while providing the necessary financial resources.

